# Troubles of Journalism

**Published:** 1881-03

**Authors:** 


TROUBLES OF JOURNALISM.—The position of editor of a medical journal, is not a bed of roses by long odds, independently of the mental and manual labor necessary to bring out a readable journal, even under favorable circumstances; the printers must be followed, and hurried up with their work, from time to time; and if they should be indifferent spellers, or at all careless to observe the rules of orthography, the types are made to call up visions, not of bright skies, and smooth and placid seas, for the editorial bark! Communications come for him to decipher, if he can, from all sorts of contributors, who write all sorts of hands, and spell in all sorts of ways. Then, all sorts of readers are to be catered for, and all sorts of comments expected from them. Some are indulgent, and forgiving, or overlook his errors and shortcomings, while others deprecate his labors, or abuse him for want of taste, or ability to prepare such matter as their intellectual appetites delight to feed upon. These are some of the troubles of journalism ; but there is another one of considerable magnitude to him, in the aggregate, though of small importance to the individual subscriber, when a few thousand of those he has striven to please for a year or more, forget, or fail to forward the subscription money, and he is compelled to cast about from issue to issue, for the ways and means of paying printer's bills. While we have probably been as fortunate as most of our cotemporaries, the delinquents on our list could lift a considerable weight from our shoulders by sending forward the dollars, and thus render us *happy*. Let them try the experiment at once, and see how we look when we are happy.

